# A roadmap for a comprehensive control of cervical cancer in Poland: integration of available solutions into current practice in primary and secondary prevention

**DOI:** 10.1097/CEJ.0000000000000528

**Published:** 2019-09-11

**Authors:** Andrzej Nowakowski, Marc Arbyn, Maryla H. Turkot, Paulina Wieszczy, Kinga Miłosz, Michał F. Kamiński, Joanna Didkowska, Mariusz Bidziński, Włodzimierz Olszewski, Mirosław Wielgoś, Maciej Krzakowski, Ernest Kuchar, Jan Walewski

**Affiliations:** a Central Coordination Center for Cervical Cancer Screening Program, Department of Cancer Prevention, Maria Sklodowska-Curie Institute-Oncology Center, Warszawa; b 2nd Department of Gynecological Oncology, Saint John of Dukla Lublin Regional Cancer Center, Lublin; c Sciensano, Unit of Cancer Epidemiology/Belgian Cancer Centre, Brussels, Belgium; d Department of Gastroenterology, Hepatology and Oncology, Medical Center for Postgraduate Education, Warsaw; e Department of Gastroenterological Oncology, Maria Sklodowska-Curie Institute-Oncology Center, Warsaw; f Department of Health Management and Health Economics, University of Oslo, Oslo, Norway; g Department of Epidemiology and Cancer Prevention; h Gynecologic Oncology Department; i Department of Pathology and Laboratory Diagnostics, Maria Sklodowska-Curie Institute–Oncology Center, Warsaw; j 1st Department of Obstetrics and Gynecology, Medical University of Warsaw, Warsaw; k Lung and Thoracic Tumors Department, Maria Sklodowska-Curie Institute-Oncology Center, Warsaw; l Department of Pediatrics with Clinical Assessment Unit, Medical University of Warsaw; m Department of Lymphoid Malignancies, Maria Sklodowska-Curie Institute–Oncology Center, Warsaw, Poland

**Keywords:** cancer screening, cervical cancer, human papillomavirus testing, human papillomavirus vaccines, liquid-based cytology, Pap smear, primary prevention, secondary prevention

## Abstract

In Poland, cervical cancer incidence and mortality still remain considerably higher than in Western European countries or North America. Recent data indicate decreasing trends in women younger than 60 years and stable trends in older women. In this article, we identified obstacles in primary and secondary prevention of cervical cancer in Poland. We analysed local legislation, management structure and organization of cervical cancer prevention in Poland and reviewed solutions available and implemented in other European countries. The main weaknesses include: (i) very low coverage of organized screening; concurrent unregistered opportunistic screening with unknown coverage and high test consumption (ii) suboptimal quality assurance in organized screening and no external quality assurance in opportunistic screening (iii) very low coverage of human papillomavirus vaccination that is not centrally reimbursed (iv) absence of pilot evaluation of (a) interventions that may improve population coverage and (b) performance of new preventive strategies. The proposed solutions are multifaceted and involve: (i) legislative and organizational regulation of cervical cancer screening aimed at comprehensive registration of procedures, data access and quality assurance (ii) pilot testing and implementation of new ways to increase coverage of cervical cancer screening, in particular among older women (iii) pilot evaluation with possible introduction of human papillomavirus-based screening and (iv) inclusion of human papillomavirus vaccination into the reimbursed national immunization program.

## Introduction

Currently, both primary and secondary prevention of cervical cancer (CC) is available to a varying degree around the world. Primary prevention through vaccination against human papillomavirus (HPV) which is the main etiological factor of CC, has been introduced into immunization programs in most of the European countries (Bruni *et al.*, 2016). Secondary prevention through early detection and treatment of cervical precancer is available in a great majority of European countries, but its effectiveness varies considerably depending on coverage, quality and organizational aspects of screening (Altobelli and Lattanzi (2015)).

In Poland, secondary prevention by cytological screening has been present for around four decades as an opportunistic intervention and since 2006/2007 as an organized screening program recommended by the European Union (Arbyn, 2008). We have recently shown decreasing trends in CC incidence and mortality in the country with a slight acceleration of the downward trends around the time of introduction of the organized screening in women at the screening age ranges. However, in women aged 60 years and older, CC incidence and mortality trends became stable (Nowakowski *et al.*, 2017). Unfavourable changes affected the organized CC screening program in Poland in 2016 which may hamper its effectiveness and requires action.

Three anti-HPV vaccines (the bivalent Cervarix, the quadrivalent Silgard and the nonavalent Gardasil 9) are registered and available in Poland. Primary prevention utilizing HPV vaccination is recommended in the Polish Preventive Vaccination Plan for girls and boys before sexual initiation and according to the schedule recommended by manufacturers. Vaccines are available free of charge for teenage girls/children only in some preventive programs run by local authorities. Because of its high out-of-pocket costs, private HPV vaccination is carried out in Poland to a minimal extent. Local programs are organized but their coverage probably does not exceed 10% in the 12–14-year-old cohort. Precise data are not available due to lack of a dedicated registry and lack of mandatory reporting from local programs.

In this roadmap, we want to identify obstacles in prevention of CC in Poland and propose solutions to improve the current situation.

## Organizational aspects of current screening for cervical cancer in Poland

### Current practice

Cytology-based CC screening became available in Poland in the 1980s. Locally organized programs were first introduced in the 1990s (Wronkowski *et al.*, 1993). Countrywide organized screening program was introduced in 2006/2007 with a target age group 25–59 years of age and a 3-year screening interval (Nowakowski *et al.*, 2015). The Program was coordinated by 16 regional and a central coordination center which: (1) mailed personal invitations for screening tests; (2) were responsible for quality assurance activities; (3) followed up women with abnormal test results; (4) organized awareness-raising activities and (5) completed other administrative and logistic tasks. The program adheres only partially to European Guidelines concerning policy and organization (Nowakowski *et al.*, 2015). From 2007 to 2015, personal invitations for screening Pap tests were posted to all eligible women registered within General Practitioners’ patient lists. By the end of 2015 mailing of personal invitations was stopped by the decision of the Ministry of Health due to considerable costs, questioned effectiveness and legal uncertainties regarding access to personal data. From 2016 on, regional coordinating centers were closed and central coordination center activities were limited to some aspects of quality assurance and training of program personnel (Table 1).

**Table 1:**
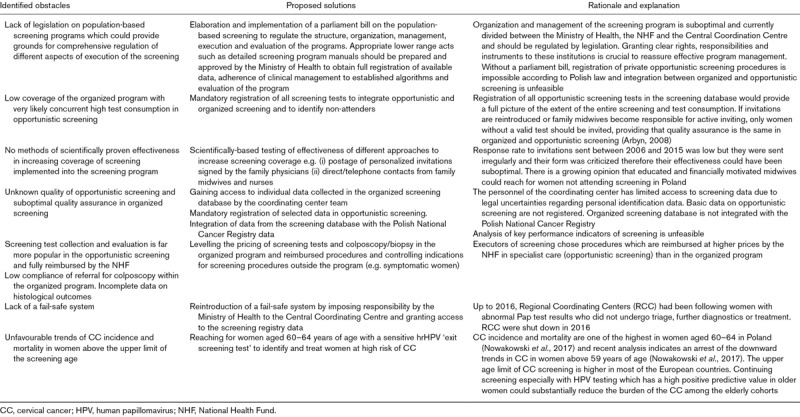
Identification of organizational and logistic obstacles in cervical cancer screening in Poland, proposed solutions and rationale for their introduction

Program triage algorithms for women with abnormal Pap smears include repeated Pap-testing (for atypical squamous cells of undetermined significance, low grade squamous intraepithelial lesion (LSIL)) or colposcopy with/without biopsy (for LSIL, atypical squamous cells-cannot exclude high grade lesion, high grade squamous intraepithelial lesion, atypical glandular cells), were set up in 2006 and have remained unchanged. HPV testing is not included in program algorithms but may be reimbursed within limited ambulatory procedures in gynecological clinics. All procedures (Pap tests, colposcopies, biopsies) performed in the program are registered in a screening database, but the coordination centre has limited access to individual data of screened women due to lack of legislative regulations and organizational problems between the coordination centre, the National Health Fund (NHF), the Ministry of Health and technical limitations of the screening database. Opportunistic Pap tests, colposcopy/biopsy procedures performed in reimbursed specialist care are priced at higher rates than in the program, therefore are favored by physicians but are not registered in the screening database. Codes of these procedures are recorded in electronic systems of the NHF, but their results are not linked to the screening database which hampers comprehensive evaluation of the entire reimbursed screening. Private opportunistic screening is widespread and very popular but not recorded due to lack of legislative obligation. Basic data on screening cohorts and coverage are presented in Table 2.

**Table 2:**
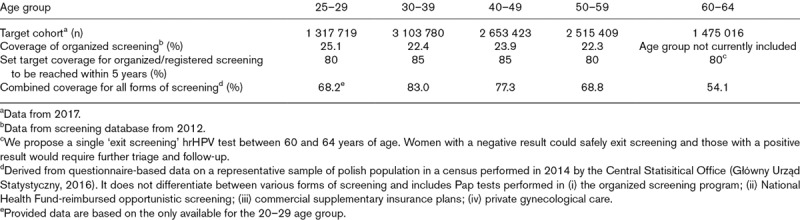
Basic data on cervical cancer screening target cohorts and coverage in Poland

### Obstacles and possible solutions

Currently in Poland there is no parliament bill which establishes the legal basis for a comprehensive regulation of execution of population-based screening programs. There are some fragmentary regulations but they do not define the responsibilities and do not grant sufficient rights to stakeholders involved in screening. Mandatory registration and linkage of required data are currently impossible. A recent inquiry identified the need for a national legislative act on the implementation of population-based screening also in Poland (Turnbull *et al.*, 2018a; Turnbull et al., 2018b; Májek *et al.*, 2019).

Low coverage of the organized program ranging from 21% to 27% without an increasing trend is the major obstacle for effective CC screening in the country (Nowakowski *et al.*, 2015). At the same time, the number of recorded (but in other systems than the screening database) opportunistic Pap tests both within and outside the target screening population was 1.7-fold higher than within the program in 2012. Audits only of cytological laboratories working in the program performed by the central coordination center provide solid data on massive test consumption (~2 mln) outside the program (Turkot *et al.*, 2018). Moreover, questionnaire-based data indicate that ~70% of women aged 20–69 had undergone Pap testing within the previous 3 years which indicates possible high-scale private-based opportunistic screening (Główny Urząd Statystyczny, 2016) (Table 2). We propose target screening coverage levels to be reached within 5 years (Table 2). In order to do it, full registration of organized and opportunistic screening tests is necessary. It will allow for quantification of over-screening and identification of women not participating in the screening (Table 3).

**Table 3:**
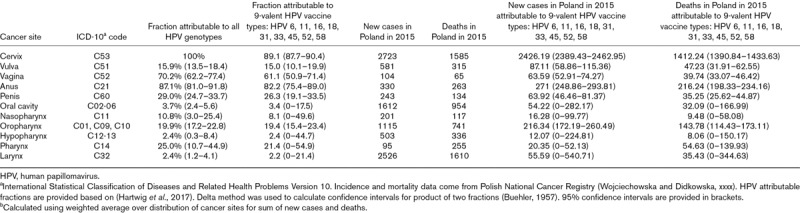
Estimated burden of cancers attributable to human papillomavirus in Poland

Postage of personal invitations for cytology was stopped in 2015 by the Ministry of Health due to questioned effectiveness and concerns about access to personal data. However outlook of invitations and irregularity of mailing could have been responsible for their low effectiveness. There are opinions among experts and societies in Poland that trained and financially motivated family midwives and nurses could improve coverage of screening but this hypothesis should be scientifically verified in a pilot study in the screening program. We therefore propose scientifically based testing of selected approaches involving personal invitations and direct contacts from financially motivated (by a success-fee) personnel of family medicine centers to reach non-attenders. Additional fees for GPs who achieve a target coverage level among their patients should also be considered. In the UK, financial incentives for doctors have been shown to be effective in improving coverage since 1993. A coverage of over 80% was reached and maintained since instalment of an organized call-recall program involving target payments whereas before 1988 the coverage was (Anttila *et al.*, 2015). In Canada, screening rates were compared between different family medicine practice models and were highest in those eligible for incentives (Pendrith *et al.*, 2016).

In 2016 and 2017 quality assurance of the program was limited to on-site audits of the clinics where Pap smears are collected, of the cytological laboratories and colposcopy centers (Turkot *et al.*, 2018). Lack of full registration and access to data on the screening process collected in the screening registry limited evaluation of the key performance indicators of the program. Low compliance to registered colposcopy/biopsy in the program (Nowakowski *et al.*, 2015; Turkot *et al.*, 2018) results in incomplete data on histological outcomes in women with abnormal Pap test results since there is no central pathology database in the country. Suggested initiatives (Table 2) tend to establish a system for quality assurance of the program. If HPV-based screening is introduced in the future, quality assurance measures should be in place as well.

Despite recommendations (Arbyn, 2008), a fail-safe mechanism to ensure that all women with positive test results are appropriately followed up have not been functioning since 2016, and its reintroduction is proposed (Table 2). Also, the issue of non-screened/under-screened older women who are at very high risk of invasive disease in Poland (Nowakowski *et al.*, 2017), has not been resolved (Table 3) although it is among the main policy recommendations of the EU guidelines (Arbyn, 2008). Reaching for these women with a sensitive hrHPV ‘exit test’ is crucial to identify women at high risk of development of CC and to protect them through timely identification and treatment of preinvasive neoplasia/early stage cancer and to decrease the burden of CC in Poland.

## New technologies in cervical cancer screening

### Human papillomavirus-based cervical cancer screening

Persistent infection with hrHPV is a cause of a large majority of CC cases worldwide (de Sanjose *et al.*, 2010). Development of invasive CC is preceded by many years by a progression of HPV-driven precancerous cervical lesions. Cervical precancer and early-stage carcinoma can be detected by exfoliative cytology which prevents fully invasive cancer.

hrHPV tests can detect CIN2+ earlier and with a sensitivity that is 20%–50% higher than sensitivity of cytology (Arbyn *et al.*, 2012). Moreover, randomized trials and screening cohort studies have demonstrated a lower incidence of invasive CC after a negative hrHPV DNA test compared to after a negative conventional or liquid-based cytology result (Arbyn *et al.*, 2012; Ronco *et al.*, 2014; Gage *et al.*, 2014).

Cross-sectional specificity of hrHPV tests especially in young women (in whom transient hrHPV infections are very common, short-lasting and regressing without squeals) is lower than that of cytology; therefore hrHPV test is not recommended for screening of women younger than 30–35 years of age (Anttila *et al.*, 2015). Adequate triage of hrHPV-positive women, restriction of HPV screening to women ≥30 years of age, lengthening of the screening interval can reduce the burden of diagnostic work-up to levels comparable to that of cytology screening (Ronco *et al.*, 2014). Given the higher protection against cancer, longer-screening intervals, the continuing decrease in cost of HPV tests makes HPV-based screening more cost-effective than cytology-based screening.

As recommended by EU guidelines (Arbyn, 2008), the introduction of a new screening strategy (e.g. with the use of a new screening test) should be preceded by pilot studies. hrHPV test should be used only in organized, population-based programs and are not recommended in opportunistic screening (Anttila *et al.*, 2015). HPV testing should begin at the age of 30–35 and stop at age 60 or 65 (provided the patient has had a recent negative test). Five years or longer screening intervals (in women with negative test results) are recommended. Only clinically validated hrHPV assays, that have demonstrated good reproducibility and non-inferior accuracy compared to a standard comparator test, should be used for CC screening (Meijer *et al.*, 2009; Anttila *et al.*, 2015; Arbyn *et al.*, 2015).

Any screening program has to include triage, referral and repeat testing for patients with a positive test result. Despite of strong recommendations to introduce HPV testing as a primary tool for screening, there are still debates on co-testing (cytology + HPV) and triage of positive HPV test results (Cuschieri *et al.*, 2018). Several triage strategies are evaluated, planned for use or used in selected countries (Cuschieri *et al.*, 2018). Reflex cytology is the most common first step to triage hrHPV-positive women and it is used routinely in Turkish (Gultekin *et al.*, 2018) and Dutch screening programs (Veen, 2017). hrHPV-positive cytology-negative women require a second triage test 6–12 months later (Anttila *et al.*, 2015). Certain countries (USA, Australia and New Zealand) include genotyping for HPV16/18 in their triage algorithms. Other markers such as p16/Ki67 immuno-chemistry and methylation markers are being evaluated. The purpose of triage is to manage women according to their risk of CIN3. A negative triage algorithm should reduce this risk under a threshold considered as sufficiently safe (<2% or <0.5%) and a positive triage should rise this risk over a level where referral is justified (for instance with a positive predictive value (PPV) of >10%) (Arbyn *et al.*, 2017).

hrHPV tests have replaced cytology as a primary screening test in Turkey (Gultekin *et al.*, 2018), the Netherlands (Veen, 2017), Australia (Cervical Cacner Screening in Australia), are recommended in the USA (Force, U.S.P.S.T.F.) and are planned for implementation or already gradually implemented in other Western/Nordic European countries (UK, Sweden, Finland) performing organized cervical screening (Changes to Cervical Cancer Screening, 2016; Ponti *et al.*, 2017).

### Liquid based cytology

Liquid based cytology (LBC) is based on a collection of cervical cells from the cervix using a special spatula or a brush and placing them in preservative solution instead of a glass slide (as in case of conventional cytology) (Koliopoulos *et al.*, 2017). After special preparation, the cells are placed in a thin layer on a slide, stained and evaluated under microscopy. Data on the accuracy of LBC vs. conventional cytology are varying. Some reports indicate that the cross-sectional sensitivity of LBC to detect CIN2+ or CIN3+ is not significantly higher than conventional cytology and the specificity tends to be slightly lower (Arbyn *et al.*, 2008; Siebers *et al.*, 2009). A recent Cochrane review revealed that pooled sensitivity of LBC is slightly higher, and pooled specificity is lower than those of conventional cytology (Koliopoulos *et al.*, 2017). LBC provides important logistical advantages: lower content of blood and inflammatory cells on slides enables shortening the time of slide evaluation and less frequent referral for repeated testing (Arbyn *et al.*, 2010; Simion *et al.*, 2014). What is important, LBC may be performed as a reflex test in case of positive HPV results from the same sample. The recent recommendation of the Polish Health Technology Assessment and Tariff Classification Agency in Poland on the reimbursement of LBC as a primary test in organized screening program is negative due to several reasons (AOTMiT, 2018).

### Proposed solutions on implementation of new technologies in cervical cancer screening in Poland

Since many countries are planning or implementing hr HPV-based screening and the Supplement to European Guidelines for Quality Assurance in CC Screening strongly advocates the use of HPV testing in organized screening only, with mandatory pilot testing before implementation (Anttila *et al.*, 2015), we have decided to assess its feasibility and performance in Poland in a randomized healthcare policy study. We consider this is the best way to generate robust data both for health technology assessment and for future clinical decision making. The study will be covered by good registration and will compare performance of the current standard (cytology predominantly conventional) with HPV-based screening and LBC triage. Currently the is a debate going on the most optimal triage strategy in HPV-based screening and different algorithms are tested or gradually implemented. In our local Polish conditions we have decided to test efficacy of the algorithm adopted in the Netherlands that has been demonstrated to be efficacious. Future changes to triage strategies are of course possible when international data prove superiority of alternative methods such as HPV genotyping or LBC based p16/Ki67. In our opinion novel triage strategies should be tested and implemented first in countries with properly functioning HPV-based screening. Primary end-points of our study will include: (1) relative detection rates of CIN2+ and CIN3+; (2) screen-test positivity; (3) PPV for CIN2+ and CIN3+ in each arm; (4) relative test-positivity; (5) PPV ratio in the HPV vs. cytology arm; and (6) burden and cost of follow-up (triage testing, colposcopy and histology, treatment). Some laboratories in Poland and other countries offer very low-cost HPV tests. Their use could largely limit the costs of screening in the country, but they require a thorough validation (Meijer *et al.*, 2009). A decision on the incorporation of new screening modalities and selection of appropriate triage/diagnostic work-up into the organized program should be made on the grounds of the results of this randomized policy trial in combination with cost-effectiveness analyses that will incorporate also international data. The trial and subsequent health technology assessment should be accompanied with international experts.

## Implementation of primary prevention as a part of the immunization program

### Introduction

HPV is responsible for virtually all cases of CC and genital warts but also for a varying proportion of cancers of the vulva, vagina, anus, penis, head and neck (Hartwig *et al.*, 2017). In Table 3 we present an estimated burden of cancers diagnosed in Poland and attributable to the most common HPV types included in the nine-valent vaccine.

Three prophylactic vaccines are currently approved for the prevention of HPV-related disease. They are safe and highly effective in prevention of neoplasia and other lesions caused by HPV-types included in them if administered before infection (Arbyn *et al.*, 2018). Population-based data are gradually acquired confirming their impact on the burden of HPV-related lesions (Nowakowski *et al.*, 2018). Taking into consideration high coverage (over 90%) of mandatory vaccination in Poland and the high efficacy of HPV vaccines if administered to adolescents, implementation of universal HPV immunization in Poland could save up to around 2000 lives a year (Table 3) after several decades needed to observe the full effect of vaccination.

The vaccines are registered in most countries in the world and are included in immunization programs in around 80 of them (Nowakowski *et al.*, 2018; Brotherton and Bloem, 2018). The programs are based on vaccine delivery in schools, primary and other healthcare centers or as a mixed approach. Vaccination coverage ranges from 8% to 98% depending on the country, type of program execution and many other factors (Nowakowski *et al.*, 2018; Brotherton and Bloem, 2018). HPV vaccines are not incorporated into the routine free-of-charge mandatory immunization schedule in Poland. WHO recommends HPV vaccination for girls aged 9–14 years with inclusion of boys and with catch-up vaccination up to the age of 18 years if funds are available (WHO, 2017).

### Proposed solutions

To follow standards of around 80 countries worldwide and over 30 in Europe and to further decrease the burden of CC and other HPV-related diseases in Poland, HPV vaccines should become a part of the routine immunization program. Bearing in mind high coverage of immunization with other paediatric vaccines in the national immunization program in Poland, the introduction of HPV vaccines into the routine free-of-charge vaccination schedule should result in high coverage. In Poland likely target age group with a two-dose schedule of HPV vaccine are 14-year-old children. This age would facilitate immunization as a result of co-administration with another prophylactic vaccine (tetanus, diphtheria and pertussis vaccine). Catch-up HPV vaccination up to the age of 18 recommended by WHO might be difficult to implement due to high costs and low experience with execution of catch-up vaccination in this age group in Poland. Further communication between experts, the Ministry of Health and Chief Sanitary Inspector are required for a prompt decision on reimbursement of HPV vaccines.

Cost-effectiveness of the three HPV vaccines may differ according to local country-specific conditions and product pricing (Brotherton and Bloem, 2018). Although there are some preliminary data on the cost-effectiveness of HPV vaccination in Poland (Berkhof *et al.*, 2013) a thorough analysis including the new nine-valent vaccine should be performed by the Polish Health Technology Assessment and Tariff Agency to select the most cost-effective product and strategy (girls/sex neutral).

## Conclusion

**Fig. 1:**
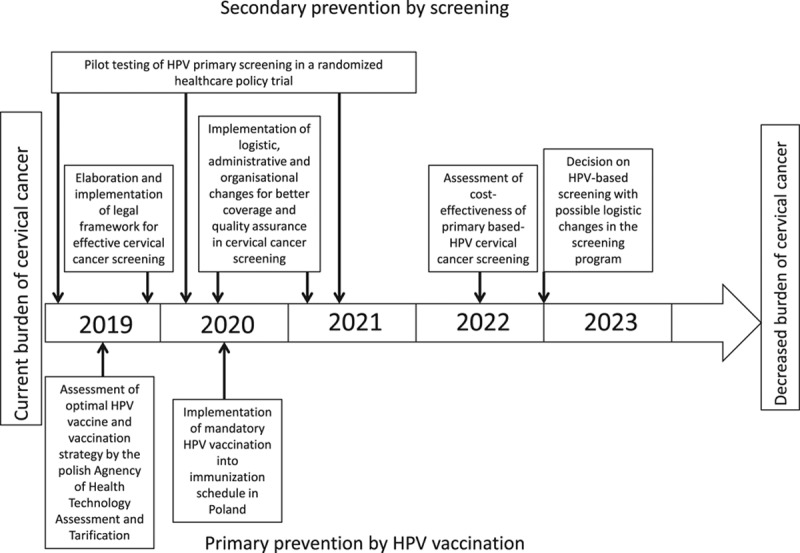
Proposed scheme of actions on the time axis to decrease the burden of cervical cancer: a roadmap for a comprehensive control of cervical cancer in Poland.

We advocate the following steps presented schematically in Fig. 1 to be undertaken to reduce the burden of CC in Poland:

(1) Initiation of legislative actions aiming at improving the organizational, logistic and administrative aspects of CC screening in Poland
(2) Execution of studies on the effectiveness of different approaches e.g. invitations vs. direct contacts of the personnel of family medicine centers on increasing of participation in screening to select and implement the most effective strategy countrywide
(3) Execution of the randomized policy study comparing current standard of cytology with HPV-based screening to provide bases for implementation of the HPV-based screening in the country
(4) Execution of a cost-effectiveness analysis of the three available HPV vaccines to select the most appropriate product and vaccination strategy for implementation in Poland
(5) Cooperation of stakeholders such as the Ministry of Health, the Chief Sanitary Inspector, Scientific and Professional Bodies to promptly introduce the selected product into the Immunization Program in Poland as a mandatory vaccine for 14-year-old girls/children

## Acknowledgements

We thank George W. Handy, Managing Director, Activity for Innovation and Economic Growth for coordination of writing of this policy article. We also thank Dr. Mark Schiffman from the National Cancer Institute in the US for a critical review and comments on the manuscript. MA was supported by the COHEAHR Network (grant no. 603019), funded by the 7th Framework Programme of DG Research and Innovation, European Commission (Brussels, Belgium), and by the Belgian Cancer Foundation.

## Conflicts of interest

There are no conflicts of interest.
